# Pulmonary metastasectomy and survival in osteosarcoma: a systematic review and meta-analysis of surgery-related prognostic factors

**DOI:** 10.1186/s12957-026-04352-0

**Published:** 2026-04-27

**Authors:** Binyuan Ning, Xiaoting Luo, Gejin Wei, Wenyu Feng, Zhuan Zou, Shanhang Li, Liang Xiong, Jifeng Miao, Haijun Tang, Feicui Li, Jianming Hu, Jingxin Deng, Sanmao Liu, Yun Liu, Qingjun Wei

**Affiliations:** 1 Department of Trauma Orthopedics and Hand Surgery, The First Affiliated Hospital of Guangxi Medical University, Nanning, China; 2 Department of Pharmacy, The First Affiliated Hospital of Guangxi Medical University, Nanning, China; 3 Department of Orthopedics, 923rd Hospital of PLA, Nanning, China; 4 Department of Bone and Joint Diseases Surgery, The Second Affiliated Hospital of Guangxi Medical University, Nanning, China; 5 Department of Spine and Osteopathic Surgery, The First Affiliated Hospital of Guangxi Medical University, Nanning, China; 6 Department of Bone and Soft Tissue Tumor, Guangxi Medical University Cancer Hospital, Nanning, China; 7 Department of Spine Surgery, Center for Orthopaedic Surgery, Orthopaedic Hospital of Guangdong Province, Academy of Orthopedics, The Third Affiliated Hospital of Southern Medical University, Guangzhou, China; 8 Medical Experimental Center, The Fifth Affiliated Hospital of Guangxi Medical University, Nanning, China

**Keywords:** Osteosarcoma, Pulmonary metastasis, Metastasectomy, Systematic review, Meta-analysis

## Abstract

**Background:**

Pulmonary metastasis is a major cause of disease progression and mortality in patients with osteosarcoma. The survival impact of pulmonary metastasectomy and the prognostic relevance of surgery-related factors remain incompletely defined. This systematic review and meta-analysis aimed to evaluate the association between pulmonary metastasectomy and survival outcomes and to identify key prognostic determinants.

**Methods:**

PubMed, Embase, Web of Science, and the Cochrane Library were systematically searched from inception to December 2025. Studies enrolling patients with histologically confirmed osteosarcoma and lung metastases that examined associations between surgery-related factors and survival outcomes were eligible. Outcomes included post-relapse survival (PRS) and post-metastasectomy overall survival (PMOS). Hazard ratios (HRs) with 95% confidence intervals (CIs) were pooled using fixed- or random-effects models according to between-study heterogeneity.

**Results:**

Twenty-one retrospective studies were included. Pulmonary metastasectomy was associated with improved post-relapse survival compared with no metastasectomy (HR = 0.29, 95% CI: 0.18–0.46). Complete resection consistently demonstrated a favorable association with survival across endpoints (PRS: HR = 0.21, 95% CI: 0.12–0.38; PMOS: HR = 0.31, 95% CI: 0.23–0.42). Bilateral pulmonary metastases were associated with inferior PMOS (HR = 1.56, 95% CI: 1.27–1.93), whereas the association with PRS did not reach statistical significance (HR = 1.37, 95% CI: 0.92–2.04). Video-assisted thoracoscopic surgery was associated with a modestly increased risk of mortality (HR = 1.69, 95% CI: 1.01–2.82). Across studies, a higher number of metastatic nodules was consistently linked to worse survival, although quantitative synthesis was limited by heterogeneous cutoff definitions.

**Conclusion:**

In observational studies, pulmonary metastasectomy is associated with improved survival in selected patients with osteosarcoma lung metastases, particularly when complete resection is achieved. Metastatic distribution, nodule burden, and surgical approach may influence prognosis. These findings support careful patient selection and individualized surgical decision-making while underscoring the need for prospective validation.

**Supplementary Information:**

The online version contains supplementary material available at 10.1186/s12957-026-04352-0.

## Introduction

Osteosarcoma is the most common primary malignant bone tumor in children and adolescents. The incidence is approximately 4.4 cases per million individuals aged 0–24 years [1], ranking eighth among all pediatric cancers [2]. Although notable progress has been achieved in neoadjuvant chemotherapy and surgical control of the primary lesion, overall survival outcomes remain far from satisfactory. Metastatic disease, particularly pulmonary metastasis which accounts for nearly 80% of all metastatic events [3, 4], constitutes the predominant cause of treatment failure and mortality.

In patients presenting with pulmonary metastases, treatment selection is complex and still lacks uniform agreement. Systemic chemotherapy continues to serve as the therapeutic foundation; however, durable survival with chemotherapy alone is uncommon [5]. Against this backdrop, pulmonary metastasectomy has emerged as an integral component of multidisciplinary management [6] and is widely regarded as a potentially curative option for carefully selected patients.

Even so, robust evidence regarding its definitive survival benefit, optimal patient selection, and the key determinants of surgical outcomes remains scarce and inconsistent [6]. Prior investigations have underscored macroscopically complete resection and metastatic nodule count as key prognostic variables [7, 8]; yet variations in survival definitions and statistical approaches across studies hamper cross-study comparison and clinical translation. Moreover, no consensus cutoff has been established regarding the nodule number that meaningfully alters long-term prognosis. Emerging data suggest that additional factors, including the anatomical distribution of metastases and the surgical approach, may also affect prognosis [9, 10], yet their independent prognostic value remains uncertain, and current clinical guidelines provide no definitive recommendations regarding these parameters [11].

To bridge these gaps, we conducted a systematic review and meta-analysis designed to quantitatively integrate current evidence on the relationship between surgical characteristics of pulmonary metastasectomy and survival in osteosarcoma patients with lung metastases. The aim was to clarify which procedural and patient-related parameters independently correlate with improved outcomes, thereby reinforcing the evidentiary basis for operative decision-making and advancing more tailored therapeutic strategies for metastatic osteosarcoma.

## Materials and methods

This systematic review and meta-analysis adhered to the Preferred Reporting Items for Systematic Reviews and Meta-Analyses (PRISMA) 2020 guidelines [12]. The protocol was prospectively registered in the International Prospective Register of Systematic Reviews (PROSPERO) under registration number CRD420251246971 and is accessible at: https://www.crd.york.ac.uk/PROSPERO/view/CRD420251246971.

### Search strategy and eligibility criteria

A structured search of four electronic databases (PubMed/MEDLINE, Embase, Web of Science, and the Cochrane Library) was undertaken. Records from database inception through December 5, 2025, were systematically retrieved. Two reviewers (BN and SL) independently implemented the search strategy using predefined keywords and Medical Subject Headings encompassing “osteosarcoma,” “pulmonary metastasis,” and “metastasectomy.” The complete search algorithm is detailed in the Supplementary Material.

All records identified through the search process were independently screened by the two reviewers in accordance with prespecified inclusion and exclusion criteria. Eligible studies met the following conditions: (1) included patients with histologically confirmed osteosarcoma and pulmonary metastases, irrespective of age and gender; (2) were published in English and conducted in human subjects; (3) evaluated the association between surgical factors related to pulmonary metastasectomy and survival outcomes, including overall survival (OS), post-relapse survival (PRS), or post-metastasectomy overall survival (PMOS); and (4) reported hazard ratios (HRs) with corresponding 95% confidence intervals (CIs) or provided sufficient data for their estimation. Studies were excluded if they: (1) lacked a control group; (2) did not provide full-text access; or (3) had an excessively small sample size (defined as ≤ 5 patients in any comparative group).

### Data extraction

Data extraction was performed independently by two reviewers for each eligible study (Table 1). The collected information comprised: (1) general study characteristics (first author, year of publication, study region); (2) sample sizes for each comparison group; and (3) survival-related outcomes, including 5-year survival rates, median survival times, survival endpoints (OS, PRS, PMOS), reported HRs with 95% CIs, and corresponding *p*-values.

**Table 1:** Main characteristics of studies included NA: not available; PRS: post-relapse survival; OS: overall survival, (unspecified) indicates that the starting point for the overall survival record is not provided in the original text; PMOS: post-metastasectomy overall survival

First author (year)	Country	Study Design	Survival Endpoints	Year of patients collection	NOS scores	
Samer Salah (2013) [16]	Jordan	Retrospective	PRS	2001–2012		8
Hiroyuki Tsuchiya (2002) [17]	Japan	Retrospective	PRS	NA		6
Ugo Pastorino (1991) [18]	Italy	Retrospective	PRS	1970–1988		7
S R Carter (1991) [19]	United Kingdom	Retrospective	PRS	1977–1983		6
Zhenguo Liu (2022) [5]	China	Retrospective	PRS	2004–2018		9
Allen M. Goorin (1984) [20]	United States	Retrospective	PRS	1972–1981		6
Yu-Min Huang (2009) [21]	Taiwan (China)	Retrospective	OS (unspecified)	1985–2005		7
Po Kuei Wu (2009) [22]	Taiwan (China)	Retrospective	OS (from primary tumor diagnosis)	1989–2008		7
Emilie P. Buddingh (2010) [23]	Netherlands	Retrospective	OS (from primary tumor diagnosis)	1990–2008		7
Laura Belli (1989) [24]	France	Retrospective	PRS+ PMOS	1977–1985		6
G. L. van Rijk-Zwikker (1991) [25]	Netherlands	Retrospective	PRS	1980–1990		7
Vanessa L. Mettmann (2023) [26]	Germany	Retrospective	PRS	1980–2015		8
Ugo Pastorino (2023) [27]	Italy	Retrospective	PMOS	1973–2014		9
William H. Meyer (1987) [28]	United States	Retrospective	PMOS	1968–1982		7
Joe B. Putnam (1983) [29]	United States	Retrospective	PMOS	1975–1982		7
Samer Salah (2015) [30]	Jordan	Retrospective	PMOS	2000–2013		6
Vanessa L. Mettmann (2024) [31]	Germany	Retrospective	OS (from primary tumor diagnosis)	1980–2022		7
Timothy B. Lautz (2021) [32]	North America	Retrospective	OS (unspecified)	1996–2018		7
Christopher Kuo (2023) [33]	United States	Retrospective	PRS	2004–2018		7
Najat C. Daw (2006) [34]	United States	Retrospective	OS (unspecified)	1986–1997		6
Matthew T. Harting (2005) [35]	United States	Retrospective	OS (unspecified)	1980–2000		7

*NA* not available, *PRS* post-relapse survival, *OS* overall survival, (unspecified) indicates that the starting point for the overall survival record is not provided in the original text, *PMOS* post-metastasectomy overall survival

Because most studies broadly reported “OS” while defining survival from different starting points, direct pooling was considered likely to introduce substantial heterogeneity. Accordingly, survival endpoints were standardized: studies explicitly defining survival from the diagnosis of pulmonary metastasis or recurrence were categorized as post-relapse survival (PRS), whereas those defining survival from the time of pulmonary metastasectomy were categorized as post-metastasectomy overall survival (PMOS). Unique or unspecified OS definitions were documented accordingly in the tables.

The methodological rigor of the included observational studies was independently appraised by two reviewers using the Newcastle–Ottawa Scale (NOS). The NOS provides a star-based score (range: 0–9) across three domains: selection of study cohorts, intergroup comparability, and outcome ascertainment. Studies achieving a score of six stars or higher were regarded as meeting acceptable standards of methodological quality (Table S1).

### Statistical analysis

Hazard ratios (HRs) accompanied by 95% confidence intervals (CIs) were adopted as the principal summary statistics to quantify associations between surgery-related variables and survival outcomes in osteosarcoma patients with lung metastases. For studies that did not directly report HRs and 95% CIs, these estimates were calculated from available data using established methods described by Tierney et al. [13–15]. Because effect estimates were sourced from diverse approaches, including direct reporting, individual-level datasets, reconstruction of Kaplan–Meier curves, and calculations based on summarized statistics, some methodological variability was expected. In studies presenting both univariate and multivariate models, adjusted HRs from multivariate analyses were preferentially selected for quantitative synthesis.

For studies requiring extraction from Kaplan–Meier curves, Engauge Digitizer software (version 12.1) was used for curve digitization and HR estimation. A pooled HR > 1 indicated that the evaluated factor was associated with worse survival. Results were presented using forest plots. Sensitivity analyses were performed by sequentially excluding each study to evaluate its influence on the pooled estimates. Potential publication bias was assessed visually using funnel plots.

Between-study heterogeneity was evaluated using Cochran’s Q test and the I² statistic and interpreted as low (*p* > 0.05 and I² ≤ 25%) or moderate (25% < I² ≤ 50%). Based on the degree of heterogeneity observed during pre-analysis, the appropriate effect model was selected: a fixed-effect model for low heterogeneity and a random-effects model otherwise. Statistically significance was considered whenever *p*-values < 0.05. Engauge Digitizer version 12.1 and R version 4.5.2 were used to obtain the data and analysis above.

## Results

### Literature search results

**Fig. 1:**
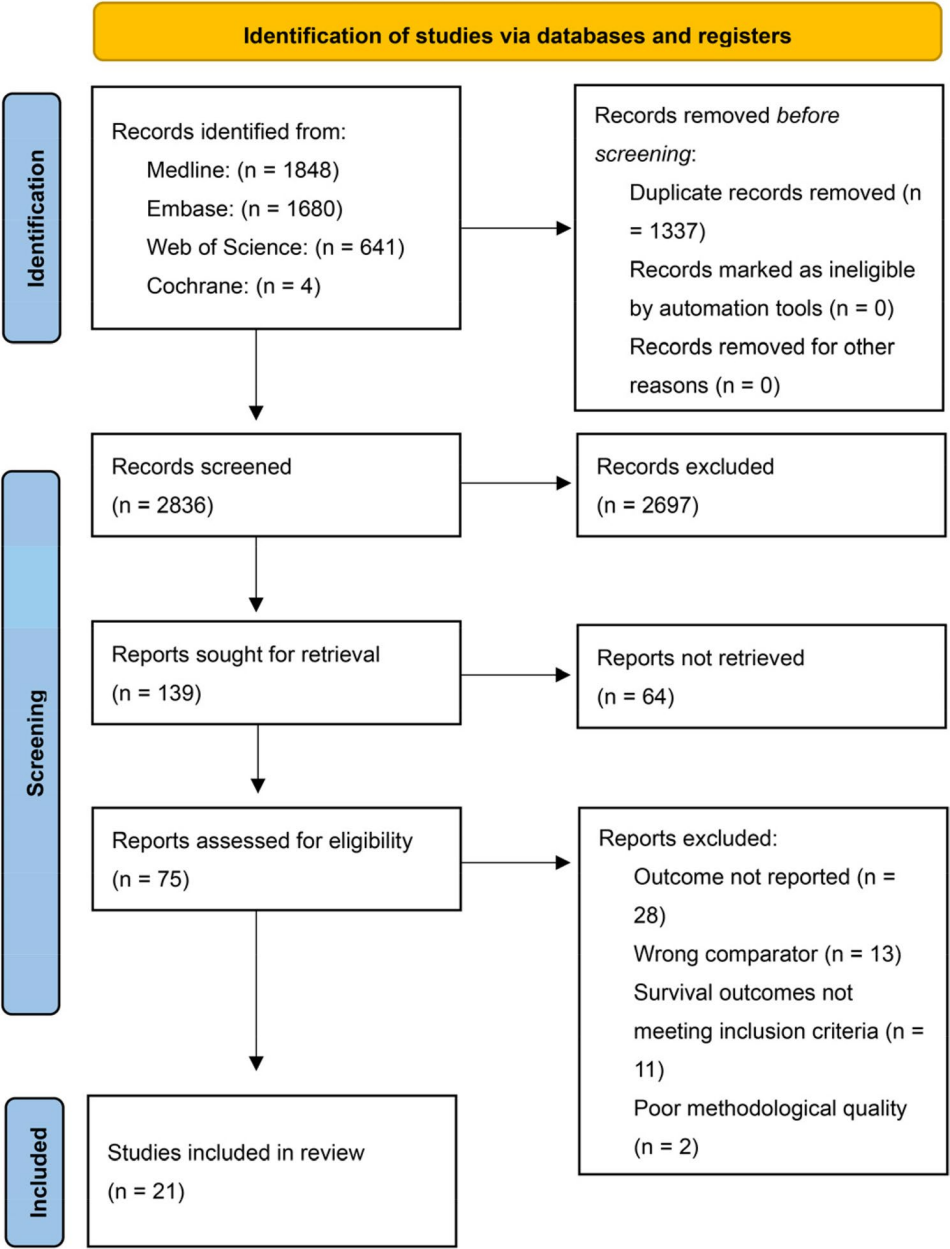
PRISMA flowchart

The systematic database search identified 4,173 records (Fig. 1). After removal of 1,337 duplicates, 2,836 records underwent title and abstract screening, and 2,697 were excluded for obvious irrelevance or failure to meet the predefined eligibility criteria, including non-osteosarcoma studies, studies unrelated to pulmonary metastases or pulmonary metastasectomy, non-human studies, and non-English publications. Of the 139 articles assessed in full text, 118 were excluded after detailed evaluation. Ultimately, 21 studies satisfied the inclusion criteria and were incorporated into the systematic review [5, 16–35]. Although certain studies originated from overlapping research groups and may have included partially shared patient populations, each provided distinct comparison cohorts for analysis; consequently, this potential overlap was not considered to materially influence the pooled estimates.

### Impact of pulmonary metastasectomy on survival

**Fig. 2:**
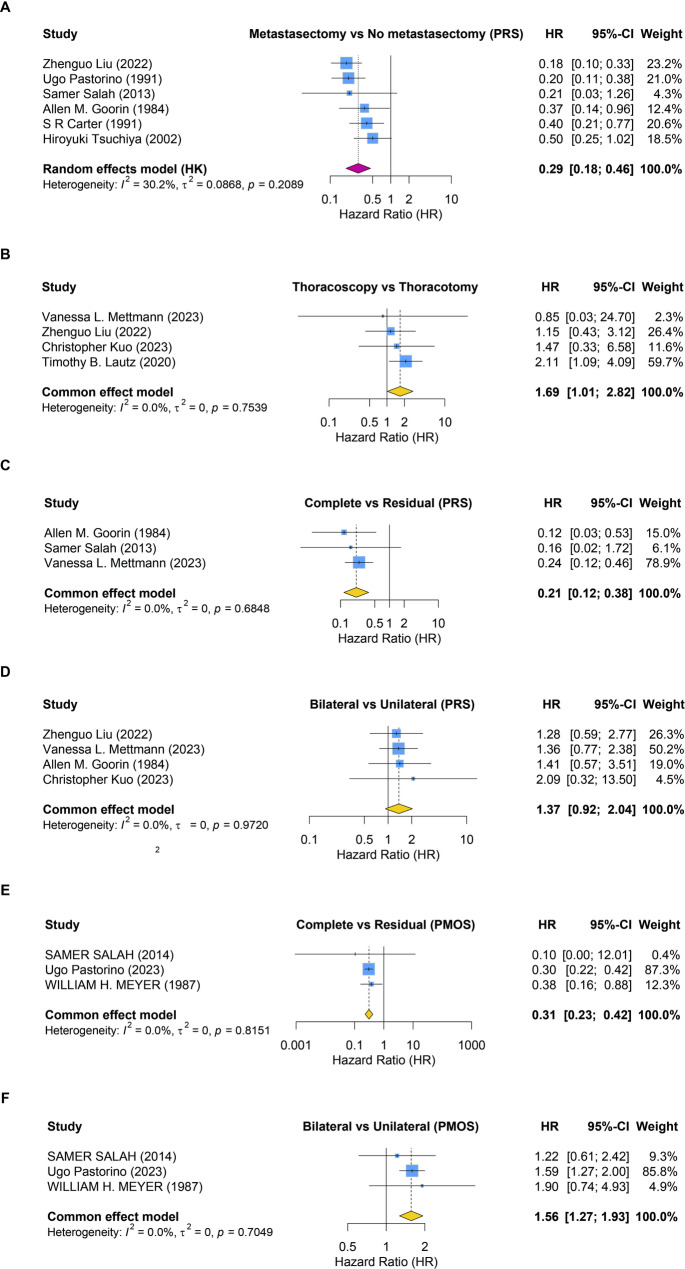
Forest plots of pooled hazard ratios for survival outcomes associated with pulmonary metastasectomy and surgical characteristics. **A** Metastasectomy versus no metastasectomy (PRS). **B** Thoracoscopy (video-assisted thoracoscopic surgery) versus thoracotomy (open surgery). **C** Complete versus residual resection (PRS). **D** Bilateral versus unilateral metastases (PRS). **E** Complete versus residual resection (PMOS). **F** Bilateral versus unilateral metastases (PMOS). PRS, post-relapse survival; PMOS, post-metastasectomy overall survival

Nine studies encompassing 722 patients examined the association between pulmonary metastasectomy and survival (Table 2). Across these reports, hazard ratios consistently favored the metastasectomy group, with values ranging from 0.18 (95% CI: 0.10–0.33) to 0.50 (95% CI: 0.25–1.02). Metastasectomy was associated with significantly improved post-relapse survival (PRS) compared with no metastasectomy in a meta-analysis of six studies (HR = 0.29, 95% CI: 0.18–0.46, *p* < 0.001; Fig. 2A). Between-study heterogeneity was moderate (I² = 30.2%, *p* = 0.209).

**Table 2:** Comparison of survival outcomes between metastasectomy and no metastasectomy with pulmonary metastases in osteosarcoma NA: not available; Rep: reported; SC: survival curve; IPD: individual participant data; Est: Estimated from summary statistics (log-rank *p* value, survival rates, SE, etc.); HR: hazard ratio; The data in parentheses represent the 95% confidence interval

Study (year)	Group	Patient (*n*)	5-year survival (%)	Median survival (Months)	Reported *p* value	HR	HR source
Samer Salah (2013) [16]	Metastasectomy	14	NA	34 (24.30 - NA)	0.0044	0.20 (0.03–1.26)	SC
	No metastasectomy	8	NA	12.39 (3.25–28.98)			
Hiroyuki Tsuchiya (2002) [17]	Metastasectomy	137	10% − 48%	NA	< 0.0001	0.50 (0.25–1.02)	SC
	No metastasectomy	85	0% − 8%	NA			
Ugo Pastorino (1991) [18]	Metastasectomy	27	47%	> 36	< 0.001	0.20 (0.11–0.38)	SC
	No metastasectomy	22	0%	8			
S R Carter (1991) [19]	Metastasectomy	25	20%	24	NA	0.40 (0.21–0.77)	SC
	No metastasectomy	18	NA	8			
Zhenguo Liu (2022) [5]	Metastasectomy	59	41%	24.9	< 0.001	0.19 (0.10–0.33)	Rep
	No metastasectomy	66	0%	13.5			
Allen M. Goorin (1984) [20]	Metastasectomy	26	NA	23.0 (18-NA)	0.0348	0.37 (0.14–0.96)	IPD
	No metastasectomy	6	NA	12.5 (6-NA)			
Yu-Min Huang (2009) [21]	Metastasectomy	24	NA	NA	< 0.05	0.47 (0.22–0.99)	SC
	No metastasectomy	28	NA	NA			
Po Kuei Wu (2009) [22]	Metastasectomy	52	45.6%	NA	0.069	0.62 (0.37–1.09)	Est
	No metastasectomy	37	28.3%	NA			
Emilie P. Buddingh (2010) [23]	Metastasectomy	56	NA, 10-year: 23%	NA	< 0.0001	0.30 (0.16–0.59)	SC
	No metastasectomy	32	NA	NA			

*NA* not available, *Rep* reported, *SC* survival curve, *IPD* individual participant data, *Est* Estimated from summary statistics (log-rank p value, survival rates, SE, etc.); *HR* hazard ratio; The data in parentheses represent the 95% confidence interval

### Impact of resection completeness on survival

Seven studies involving a total of 887 patients compared outcomes between macroscopically complete resection (CR) and residual resection (RR) groups (Table 3). Five-year survival in the CR cohorts ranged from 25% to 80.8%, whereas corresponding rates in the RR groups were substantially lower, spanning 0% to 20%. In every study, the HR for CR was below 1 (range: 0.105–0.522), indicating a consistent association with improved survival. Depending on data availability, two independent meta-analyses were subsequently undertaken. For PRS (Fig. 2C), the pooled HR from three studies was 0.21 (95% CI: 0.12–0.38, *p* < 0.001), with low heterogeneity (I² = 0.0%, *p* = 0.685). For post-metastasectomy overall survival (PMOS) (Fig. 2E), pooling four studies yielded an HR of 0.31 (95% CI: 0.23–0.42, *p* < 0.001), with low heterogeneity (I² = 0.0%, *p* = 0.815). These results support a clear survival benefit when macroscopically complete resection of pulmonary metastases is achieved.

**Table 3:** Comparison of survival outcomes between complete and residual resection of pulmonary metastases in osteosarcoma NA: not available; Rep: reported; SC: survival curve; IPD: individual participant data; Est: Estimated from summary statistics (log-rank *p* value, survival rates, SE, etc.); HR: hazard ratio; The data in parentheses represent the 95% confidence interval

Study (year)	Group	Patient (*n*)	5-year survival (%)	Median survival (Months)	Reported *p* value	HR	HR source
Laura Belli (1989) [24]	Complete	17	37%	52	NA	0.27 (0.04–1.60)	SC
	Residual	27	NA	6			
Allen M. Goorin (1984) [20]	Complete	11	80.8% (60–100%)	NA	0.0009	0.12 (0.03–0.53)	IPD
	Residual	15	13.3% (3.7–48.4%)	18.00 (11.00–40.00)			
Vanessa L. Mettmann (2023) [26]	Complete	203	53.7%	NA	< 0.001	0.24 (0.12–0.46)	SC
	Residual	11	20%	NA			
Ugo Pastorino (2023) [27]	Complete	430	NA, 20-year: 31.5% (27.0–36.0%)	27.6 (24.6–33.6)	0.015	0.30 (0.22–0.42)	SC
	Residual	33	NA, 10-year: 6.1% (1.1–17.6%)	8.4 (6.6–13.0)			
William H. Meyer (1987) [28]	Complete	24	NA	NA	0.0379	0.38 (0.16–0.89)	SC
	Residual	13	NA	NA			
Samer Salah (2015) [30]	Complete	19	38%	25	0.36	0.10 (0.00–13.08)	Est
	Residual	6	0%	29			
Vanessa L. Mettmann (2024) [31]	Complete	10	25%	NA	< 0.001	0.52 (0.36–0.77)	Est
	Residual	68	5.6%	NA			

*NA* not available, *Rep* reported, *SC* survival curve, *IPD* individual participant data, *Est* Estimated from summary statistics (log-rank p value, survival rates, SE, etc.), *HR* hazard ratio. The data in parentheses represent the 95% confidence interval

### Impact of metastasis distribution on survival

Seven studies comprising 1,311 patients evaluated survival differences between unilateral and bilateral pulmonary metastases (Table 4). Reported five-year survival rates ranged from 33% to 89% for unilateral disease and from 22.2% to 46.5% for bilateral involvement. All studies presented HRs greater than 1 for bilateral metastases (range: 1.21–2.08), indicating a tendency toward increased mortality; nevertheless, the original analyses did not reach statistical significance (*p* > 0.05).

**Table 4:** Comparison of survival outcomes between bilateral and unilateral pulmonary metastases in osteosarcoma NA: not available; Rep: reported; SC: survival curve; IPD: individual participant data; Est: Estimated from summary statistics (log-rank *p* value, survival rates, SE, etc.); HR: hazard ratio; The data in parentheses represent the 95% confidence interval

Study (year)	Group	Patient (*n*)	5-year survival (%)	Median survival (Months)	Reported *p* value	HR	HR source
Allen M. Goorin (1984) [20]	Bilateral	9	22.2%	22 (12 - NA)	0.5	1.41 (0.57–3.51)	IPD
	Unilateral	23	37.1%	26 (13 - NA)			
Vanessa L. Mettmann (2023) [26]	Bilateral	40	46.5%	NA	0.285	1.36 (0.78–2.38)	Est
	Unilateral	141	56.9%	NA			
Zhenguo Liu (2022) [5]	Bilateral	49	NA	NA	0.538	1.28 (0.59–2.77)	Rep
	Unilateral	76	NA	NA			
Christopher Kuo (2023) [33]	Bilateral	15	NA, 2-year 73% (43.6–89.1%)	NA	0.5	2.09 (0.32–13.50)	SC
	Unilateral	9	NA, 2-year 89% (43.3–98.4%)	NA			
Ugo Pastorino (2023) [27]	Bilateral	141	NA, 20-year: 17.5% (11.6–24.5%)	20.4 (15.1–24.0)	0.478	1.59 (1.27–2.00)	Rep
	Unilateral	315	NA, 20-year: 34.5% (29.2–39.9%)	29.6 (25.5–36.9)			
Samer Salah (2015) [30]	Bilateral	141	26%	29	0.58	1.22 (0.61–2.42)	Est
	Unilateral	313	33%	25			
William H. Meyer (1987) [28]	Bilateral	13	NA	NA	0.0575	1.90 (0.74–4.93)	SC
	Unilateral	26	NA	NA			

*NA* not available, *Rep* reported, *SC* survival curve, *IPD* individual participant data, *Est* Estimated from summary statistics (log-rank p value, survival rates, SE, etc.), *HR* hazard ratio. The data in parentheses represent the 95% confidence interval

Two meta-analyses were performed. For PRS (Fig. 2D), the pooled HR was 1.37 (95% CI: 0.92–2.04, *p* = 0.119), with low heterogeneity (I² = 0.0%, *p* = 0.972). For PMOS (Fig. 2F), the pooled HR was 1.56 (95% CI: 1.27–1.93, *p* < 0.001), also with low heterogeneity (I² = 0.0%, *p* = 0.705). These results indicate that bilateral pulmonary metastasis is associated with a significantly higher risk of mortality, particularly when survival is measured from the time of metastasectomy.

### Impact of surgical approach on survival

Four studies involving 471 patients compared survival between video-assisted thoracoscopic surgery (VATS) and open surgery (Table 5). Only one study explicitly reported that VATS was associated with increased mortality risk [32], whereas the remaining three studies observed no statistically significant difference [5, 26, 33]. The pooled HR was 1.69 (95% CI: 1.01–2.82, *p* = 0.044), with low heterogeneity (I² = 0%, *p* = 0.754) (Fig. 2B). This finding suggests that VATS may be associated with an elevated risk of mortality.

**Table 5:** Comparison of survival outcomes between thoracoscopic and open surgical resection of pulmonary metastases in osteosarcoma

Study (year)	Group	Patient (*n*)	5-year survival (%)	Median survival (Months)	Reported *p* value	HR	HR statistic
Timothy B. Lautz (2021) [32]	Thoracoscopy	48	42%	NA	0.027	2.11 (1.09–4.09)	Rep
	Thoracotomy	154	49%	NA			
Vanessa L. Mettmann (2023) [26]	Thoracoscopy	23	60.2%	NA	0.926	0.85 (0.03–25.33)	Est
	Thoracotomy	162	55.1%	NA			
Christopher Kuo (2023) [33]	Thoracoscopy	16	NA, 2-year: 78% (46.5–92.5%)	NA	0.59	1.47 (0.33–6.59)	SC
	Thoracotomy	18	NA, 2-year: 93% (61.3–99%)	NA			
Zhenguo Liu (2022) [5]	Thoracoscopy	39	NA	NA	0.078	1.15 (0.43–3.12)	Rep
	Thoracotomy	11	NA	NA			

*NA* not available, *Rep* reported, *SC* survival curve, *Est* Estimated from summary statistics (log-rank *p* value, survival rates, SE, etc.), *HR* hazard ratio. The data in parentheses represent the 95% confidence interval

### Impact of nodule number on survival

Thirteen studies, collectively including more than 1,300 patients, explored the relationship between pulmonary metastatic nodule count and survival outcomes (Table 6). In general, a lower number of nodules corresponded to more favorable survival, with substantially higher five-year survival rates observed in patients presenting with a solitary lesion or limited nodule burden. For patients with a solitary nodule, the five-year survival rate reached 50% [21], and one study with over 20 years of follow-up reported a 20-year survival rate of 43% (95% CI: 36.3–49.6%) [27].

**Table 6:** Comparison of survival outcomes by number of pulmonary metastatic nodules in osteosarcoma

Study (year)	No. of lung nodules	Patient (*n*)	5-year survival (%)	Median survival (Months)	Reported *p* value	HR	HR source
Allen M. Goorin (1984) [20]	= 1	13	49.9% (27.9–89.2%)	40.00 (26.00 - NA)			
	≥ 2	19	21.1% (8.8–50.3%)	18.00 (12.00 - NA)	0.0482	2.56 (0.98–6.67)	IPD
Joe B. Putnam (1983) [29]	≤ 3	32	NA	37			
	> 3	6	NA	10	0.039	4.40 (0.45–42.78)	SC
William H. Meyer (1987) [28]	< 4	28	NA	NA			
	≥ 4	9	NA	NA	0.0067	4.81 (1.66–13.89)	SC
Zhenguo Liu (2022) [5]	= 1	73	NA	NA			
	= 2	34	NA	NA	0.316	0.68 (0.32–1.45)	Rep
	= 3	18	NA	NA	0.325	0.59 (0.20–1.69)	Rep
Timothy B. Lautz (2021) [32]	< 5	138	54%	NA			
	≥ 5	49	41%	NA	< 0.01	NA	
	= 1	NA	NA	NA	0.006		
	2–3	NA	NA	NA		2.02 (0.93–4.40)	Rep
	4–9	NA	NA	NA		2.50 (1.04–5.97)	Rep
	≥ 10	NA	NA	NA		4.40 (1.85–10.46)	Rep
Ugo Pastorino (2023) [27]	= 1	223	NA, 20-year: 43% (36.3–49.6%)	49.8 (31.4–217.4)	0.058		
	2–4	151	NA, 20-year: 20.7% (14.5–27.6%)	20.5 (17.0–26.2)		1.59 (1.23–2.06)	SC
	5–9	63	NA, 20-year: 13.5% (6.3–23.5%)	19.9 (13.1–22.8)		1.94 (1.34–2.81)	SC
	≥ 10	26	NA, 20-year: 7.7% (1.3–21.7%)	20.3 (10.6–28.4)		2.38 (1.35–4.19)	SC
Po Kuei Wu (2009) [22]	< 3	41	56.5%	NA			
	≥ 3	13	21.3%	NA	0.002	2.70 (1.44–5.10)	Est
Hiroyuki Tsuchiya (2002) [17]	≤ 3	172	0–40%	NA			
	> 3	98	0–13%	NA	< 0.0001	NA	
Samer Salah (2015) [30]	< 3	12	27%	25			
	≥ 3	13	29%	24.8	0.85	0.95 (0.53–1.69)	Est
Yu-Min Huang (2009) [21]	= 1	NA	50%	NA			
	≥ 2	NA	5%	NA	0.017	NA	
Najat C. Daw (2006) [34]	≤ 3	10	40%	NA			
	> 3	13	15.4%	NA	0.014	3.15 (0.82–12.06)	SC
Matthew T. Harting (2006) [35]	≤ 5	55	32.7%	NA			
	> 5	28	17.9%	NA	0.313	1.94 (0.91–4.14)	SC

*NA* not available, *Rep* reported, *SC* survival curve, *IPD* individual participant data, *Est* Estimated from summary statistics (log-rank *p* value, survival rates, SE, etc.); HR: hazard ratio; The data in parentheses represent the 95% confidence interval

Using a cutoff of three nodules, Wu et al. reported five-year survival rates of 56.5% and 21.3% for patients with < 3 and ≥ 3 nodules, respectively [22]. Except for Salah et al. [30], similar trends were observed across studies using a cutoff of ≤ 3 nodules [17, 29, 34]. When stricter cutoffs were applied, the survival gap widened further. For instance, Goorin et al. and Huang et al. reported five-year survival rates of 49.9% versus 21.1% [20] and 50% versus 5% [21] for patients with one versus ≥ 2 nodules, respectively. By contrast, when higher cutoffs were used—such as the ≤ 5 versus > 5 classification in Harting et al.—five-year survival rates were 32.7% and 17.9%, respectively [35], with a comparatively smaller intergroup difference.

A greater number of nodules generally corresponded to higher hazard ratios. Pastorino et al. demonstrated a stepwise increase in HRs relative to a solitary lesion: 1.59 for 2–4 nodules, 1.94 for 5–9 nodules, and 2.38 for ≥ 10 nodules [27], a trend similarly described by Lautz et al. [32]. In contrast, Liu et al. found no statistically significant survival difference when comparing solitary nodules with two or three nodules, reporting HRs (95% CI) of 0.680 (0.319–1.446) and 0.588 (0.204–1.692), respectively [5]. Because definitions of nodule count cutoffs varied considerably among studies, quantitative pooling was not undertaken.

### Sensitivity analysis and publication bias

**Fig. 3:**
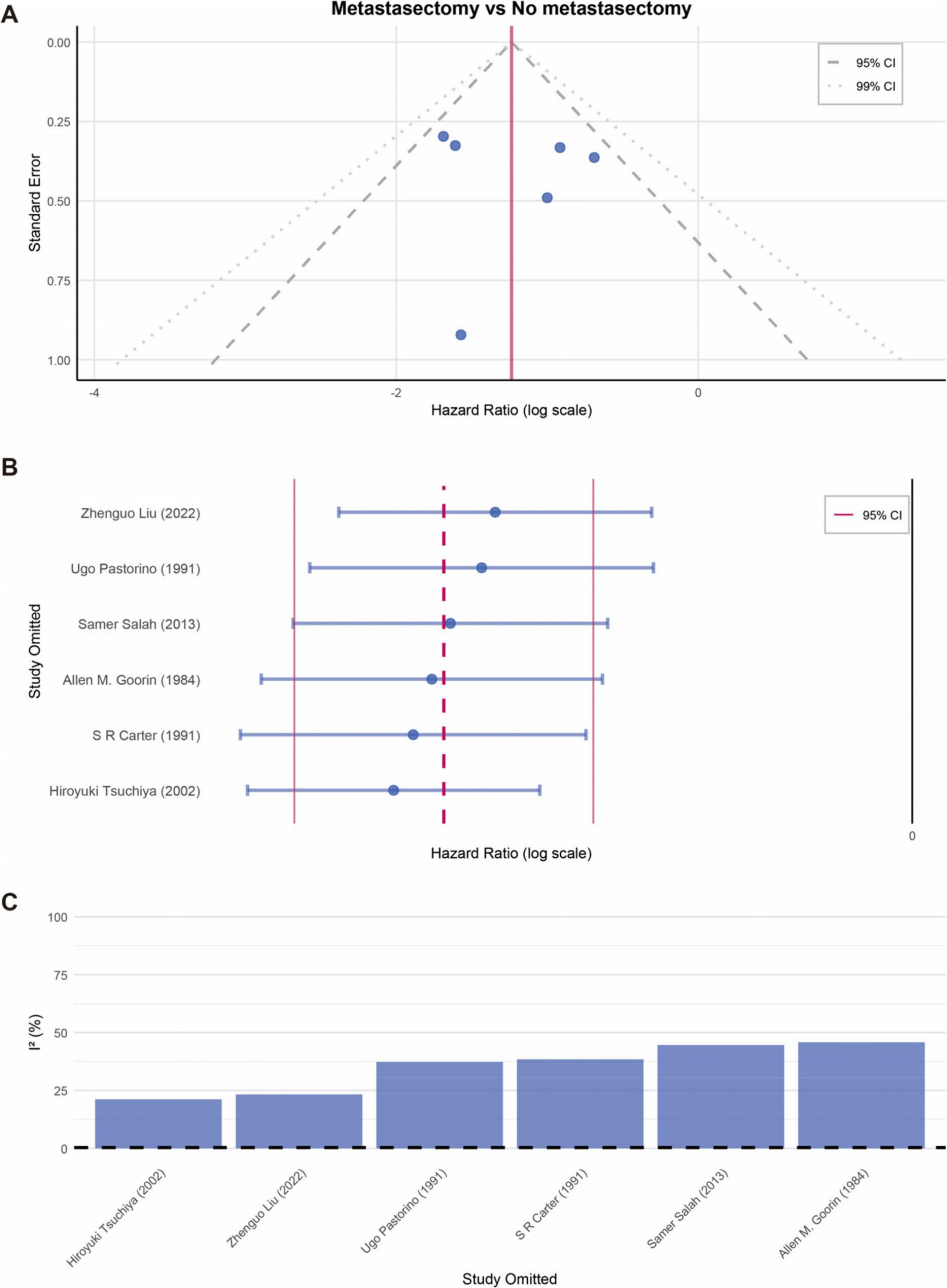
Sensitivity and publication bias analyses for metastasectomy versus no metastasectomy. **A** Funnel plot assessing potential publication bias. **B** Leave-one-out sensitivity analysis. **C** Influence analysis of heterogeneity

As each comparison included fewer than ten studies, meta-regression was not feasible due to limited study numbers, and publication bias was evaluated qualitatively through visual inspection of funnel plots. In the comparison between metastasectomy and no metastasectomy, data points were distributed in an approximately symmetrical fashion around the pooled estimate, without clear evidence of small-study effects, implying a low probability of substantial publication bias (Fig. 3A). Leave-one-out sensitivity testing showed that sequential removal of individual studies did not meaningfully change the pooled HR; all recalculated estimates were consistent with the primary result, indicating that no single study disproportionately influenced the analysis (Fig. 3B). Influence diagnostics further confirmed that excluding any one study did not materially alter heterogeneity measures, reinforcing the stability of the findings (Fig. 3C).

Because relatively few studies reported PRS and PMOS separately, these endpoints were combined in exploratory analyses to increase statistical power when evaluating complete versus residual resection and unilateral versus bilateral metastases. For the macroscopically complete resection comparison, funnel plots displayed approximate symmetry, suggesting minimal risk of publication bias (Figures S1 A, C, E). In analyses of bilateral metastases, sensitivity testing again demonstrated stable pooled estimates, and funnel plots showed no obvious asymmetry (Figures S1 B, D, F). These exploratory pooled analyses warrant cautious interpretation, as merging distinct survival endpoints may introduce additional variability. Given the limited study numbers, further subgroup analyses were not conducted.

## Discussion

In this systematic review and meta-analysis, we integrated the current body of evidence addressing surgical treatment of pulmonary metastases in osteosarcoma and quantified the relationships between pulmonary metastasectomy, surgery-related variables, and long-term survival. Taken together, the data suggest that pulmonary metastasectomy may confer a survival advantage in appropriately selected patients with resectable pulmonary metastatic disease. Prognosis was also influenced by several operative and disease-specific factors, including resection completeness, metastatic distribution, nodule burden, and surgical approach. Macroscopically complete resection was consistently associated with more favorable outcomes, whereas residual disease, bilateral metastases, multiple nodules, and video-assisted thoracoscopic surgery (VATS) tended to correlate with poorer survival. Overall, these findings provide consolidated quantitative support for pulmonary metastasectomy in appropriately selected patients and offer practical implications for surgical selection and strategy.

The pooled analysis showed that pulmonary metastasectomy was linked to a significantly lower mortality risk compared with no metastasectomy (PRS HR = 0.29). This result is in keeping with established clinical experience and expert recommendations [11], which emphasize that, when complete resection is achievable, surgery may offer the best opportunity for sustained survival—or even cure—in recurrent or metastatic osteosarcoma [16, 18, 19]. The magnitude of benefit likely reflects both direct tumor debulking and the potential enhancement of subsequent systemic therapy efficacy. Admittedly, all included studies were retrospective and thus vulnerable to selection bias. Nonetheless, the direction and magnitude of effect were remarkably consistent, and sensitivity analyses confirmed that the pooled estimates were stable. In practical terms, these findings reinforce the consideration of surgical resection whenever it is technically feasible and oncologically justified. When complete resection appears achievable, a strategy aimed at maximal clearance of metastatic disease may be reasonable, although such decisions should be individualized and interpreted in light of the inherent limitations of retrospective data [36].

Among all evaluated variables, resection completeness emerged as the most decisive prognostic determinant. Across both PRS and PMOS analyses, complete resection was associated with a marked reduction in mortality risk (HR = 0.21 and HR = 0.31). From a biological standpoint, this association is credible: residual microscopic or macroscopic disease may serve as a nidus for further dissemination and early relapse, particularly in osteosarcoma, where aggressive tumor biology and relative chemoresistance restrict effective salvage options [37]. Accordingly, the surgical goal should not be limited to cytoreduction but should prioritize full oncologic clearance, in line with recent observations by Eisenberg et al. [38]. However, in retrospective studies, the assessment of resection completeness may be limited by heterogeneous surgical documentation and the lack of standardized criteria across institutions. Another potentially relevant but insufficiently reported factor is thoracic nodal involvement. Previous studies in other tumor types have suggested that lymph node metastasis may carry prognostic significance in pulmonary metastasectomy [39], and its possible association with metastatic burden may partly confound the relationship between nodule number and survival [40–42]. It is also noteworthy that the number of nodules identified intraoperatively often exceeds those detected on preoperative imaging [29, 43, 44], suggesting that exclusive reliance on imaging may underestimate disease burden and compromise resection completeness. Comprehensive preoperative planning, coupled with meticulous intraoperative exploration, is therefore essential to maximize the likelihood of achieving complete resection. However, it should be noted that surgery may still have clinical value even in patients for whom macroscopic complete resection is not achievable. One important consideration is the distinctive tendency of osteosarcoma lung metastases to cause secondary spontaneous pneumothorax (SSP) and pleural complications. Matsuura et al. provided pathological evidence that subpleural metastatic nodules may undergo bullous change, rupture the visceral pleura, and disseminate tumor cells along the pleural surface, thereby offering a plausible mechanism for pleural seeding [45]. From a surgical perspective, this suggests that selected subpleural lesions may still warrant particular attention even when complete metastasectomy is not feasible, because resection may help reduce the risks of secondary spontaneous pneumothorax (SSP), hemothorax, and potential pleural dissemination. In osteosarcoma, clinically relevant endpoints such as the incidence of SSP or hemothorax, the need for chest tube drainage, interruptions in systemic chemotherapy, and patterns of pleural recurrence may substantially influence patient management and quality of care. Although these outcomes were not reported consistently across the included studies and therefore could not be synthesized in the present meta-analysis, they may still represent meaningful indications for metastasectomy in selected patients, even when a clear survival benefit cannot be demonstrated.

Metastatic distribution also appeared to have prognostic relevance. In our meta-analysis, bilateral pulmonary involvement was associated with worse survival in the PMOS analysis (HR = 1.56, *p* < 0.001), whereas this association did not reach statistical significance in the PRS analysis (HR = 1.37, *p* = 0.119). This discrepancy may reflect limited sample sizes and the predominance of univariate analyses in several studies. In other tumor types, bilateral involvement has been shown to reflect a greater tumor burden and more aggressive tumor biology [46], and it also poses increased technical challenges during surgical management. These factors may likewise contribute to the unfavorable prognosis observed in patients with pulmonary metastases from osteosarcoma. Bilateral disease is inherently associated with the presence of at least two pulmonary nodules, and this may partly contribute to the adverse survival trend observed in patients with bilateral pulmonary metastases. Future studies are warranted to adjust for nodule number in multivariable models to better clarify the true prognostic significance of bilateral versus unilateral pulmonary metastases. Nevertheless, most original studies did not demonstrate statistically significant differences between unilateral and bilateral disease in their individual analyses [5, 20, 26–28, 30, 33]. Given that the majority relied on univariate methods without adjustment for potential confounders, further well-designed investigations are warranted to clarify the independent prognostic role of metastatic distribution and to inform surgical decision-making.

The impact of surgical approach warrants particular consideration. Our pooled results suggested that VATS may be associated with inferior survival (HR = 1.69, 95% CI: 1.01–2.82, *p* = 0.044). Although this finding differs from the favorable experience of minimally invasive techniques in some adult solid tumors, it is biologically plausible in osteosarcoma, where VATS may limit tactile detection of deep or non-palpable nodules and thereby increase the likelihood of residual disease [47]. Given that opportunities for repeat resections are often limited in this population, even small unresected lesions may adversely affect long-term outcomes. It is noteworthy that, in some reports, patients undergoing VATS were typically those with fewer nodules and comparatively favorable disease profiles [32]. Against this background, the observed association may represent an even more concerning signal. Although VATS is associated with reduced perioperative morbidity and faster recovery, questions remain regarding its oncologic thoroughness, and any potential survival trade-offs must be weighed carefully. In addition, the interval between preoperative CT and surgery may represent an underrecognized source of bias. In an aggressive tumor such as osteosarcoma, imaging performed several weeks before resection may underestimate the true metastatic burden at the time of surgery, yet this variable was rarely reported in the included retrospective studies. Moreover, some small subpleural lesions are also difficult to detect on CT, but may still be identified intraoperatively through direct inspection and palpation. As a result, VATS may miss subtle but clinically relevant lesions, potentially increasing the risks of incomplete resection, postoperative secondary spontaneous pneumothorax (SSP), and pleural dissemination [45]. Three studies found no statistically significant differences between thoracoscopic and open procedures [5, 26, 33], whereas the largest cohort analysis aligned with the pooled result [32]. Given the small number of available studies, however, this inference should be approached cautiously. Divergent conclusions have also been reported in the literature: Passos et al. described potential advantages of VATS for pulmonary metastases [10], while Oliveira et al. and Gossot et al. observed comparable outcomes between minimally invasive and open approaches [48, 49]. Markowiak et al. and Mutsaerts et al. reported similar survival only among patients with solitary lung metastasis [50, 51]. Importantly, these investigations encompassed heterogeneous tumor types and were not confined to osteosarcoma. Therefore, selection of surgical approach in osteosarcoma pulmonary metastases warrants deliberate evaluation. An ongoing randomized controlled trial [52] is expected to clarify this issue and provide more definitive guidance on the optimal operative strategy.

Across nearly all included studies, a higher number of metastatic nodules corresponded to worse survival, underscoring tumor burden as a central prognostic factor. However, wide variation in the definition of nodule count cutoffs prevented formal meta-analysis and highlights the need for standardized criteria in future investigations. Liu et al. reported that survival among patients with fewer than four nodules was not significantly different from that of those with a solitary lesion [5], suggesting a potentially meaningful clinical benchmark. In other sarcoma subtypes, multiple pulmonary nodules may be amenable to repeated resections without dramatic survival disparities [53–55]. By contrast, osteosarcoma lung metastases are frequently characterized by multidrug resistance and limited opportunities for iterative surgery [56], and differences in nodule burden appear to translate into substantial survival variation. Establishing clinically relevant cutoff values for metastatic nodules is therefore of practical importance. Moreover, in patients with extensive nodular disease for whom complete resection is clearly unfeasible, the actual benefit of palliative surgery remains uncertain—particularly considering the typically young age and surgical resilience of this population. This question carries both clinical and ethical implications and merits further rigorous study.

Previous studies have evaluated the efficacy of pulmonary metastasectomy and associated prognostic factors in soft tissue sarcomas [49, 53, 57, 58], and broader sarcoma reviews, particularly that by Treasure et al., also summarized a limited number of studies involving osteosarcoma pulmonary metastases and highlighted the potential beneficial role of pulmonary metastasectomy [59]. However, given the younger age at onset and the particularly aggressive biological behavior of osteosarcoma, pooling osteosarcoma with other sarcoma subtypes may be inappropriate. Notably, a small subset of younger patients with osteosarcoma may have underlying Li-Fraumeni syndrome, a rare hereditary cancer predisposition syndrome associated with a substantially increased risk of multiple early-onset malignancies. This persistent lack of uniformity in the evaluation and management of pulmonary metastases also highlights the need for a more standardized framework for disease classification. Migliore et al. dedicated staging proposals for lung metastases have been suggested to facilitate a common language across centers and improve comparability among studies [40–42]. Moreover, considering the psychological burden associated with amputation in some pediatric patients with primary osteosarcoma, surgical decision-making in the setting of recurrence should be undertaken with particular caution.

In the present study, survival endpoints were distinguished according to their starting time points, and post-relapse survival (PRS) and post-metastasectomy overall survival (PMOS) were analyzed separately, thereby reducing heterogeneity arising from inconsistent definitions of overall survival (OS). Nevertheless, several limitations warrant consideration. First, all included studies were retrospective in design, and selection bias is unavoidable. Second, the sample sizes were relatively small and the number of studies available for each comparison was limited, which may have reduced statistical power, precluded a formal assessment of publication bias, and prevented evaluation of some potentially relevant prognostic factors, such as the interval from primary tumor treatment to the development of pulmonary metastases, because this variable was inconsistently defined and insufficiently reported across studies. Third, hazard ratios were derived from multiple sources—including directly reported statistics, reconstructed Kaplan–Meier curves, and aggregate data—which may have introduced methodological heterogeneity. Fourth, inter-center differences in surgical techniques, chemotherapy regimens, and follow-up strategies may have influenced the results; in particular, variability in chemotherapy protocols posed a substantial challenge to isolating the effect of surgery on survival outcomes. Therefore, the findings should be interpreted with caution.

## Conclusion

This meta-analysis indicates that, among carefully selected patients with osteosarcoma lung metastases, pulmonary metastasectomy correlates with a meaningful survival advantage. The extent of this benefit is closely tied to the attainment of complete resection. Beyond resection status, metastatic distribution, operative approach, and nodule burden also exert substantial influence on prognosis. Collectively, these findings reinforce the role of curative-intent pulmonary metastasectomy within multidisciplinary treatment frameworks. Future investigations should focus on optimizing patient selection criteria and refining surgical strategies to maximize the population likely to benefit from this aggressive intervention.

## Supplementary Information

Supplementary Material 1.

Supplementary Material 2.

## Data Availability

No datasets were generated or analysed during the current study.

## Abbreviations

CR: Complete resection
CI: Confidence interval
HR: Hazard ratio
NOS: Newcastle–Ottawa Scale
OS: Overall survival
PMOS: Post-metastasectomy overall survival
PRS: Post-relapse survival
PRISMA: Preferred Reporting Items for Systematic Reviews and Meta-Analyses
PROSPERO: International Prospective Register of Systematic Reviews
RR: Residual resection
VATS: Video-assisted thoracoscopic surgery
