# Supplementary material for: Pulmonary metastasectomy and survival in osteosarcoma: a systematic review and meta-analysis of surgery-related prognostic factors
Source: World J Surg Oncol. 2026 Apr 27;24:247. doi: 10.1186/s12957-026-04352-0 (PMC13251309; doi:10.1186/s12957-026-04352-0)
Supplement: Supplementary file 1 — Supplementary Material 1. [file 12957_2026_4352_MOESM1_ESM.docx]

**Supplementary Appendix: Detailed Search Strategies**

**PubMed/MEDLINE:**

((((((((((((osteosarcoma[Title/Abstract]) OR (Osteosarcomas[Title/Abstract])) OR (Osteogenic Sarcoma[Title/Abstract])) OR (Osteosarcoma Tumor[Title/Abstract])) OR (Osteosarcoma Tumors[Title/Abstract])) OR (Tumor, Osteosarcoma[Title/Abstract])) OR (Tumors, Osteosarcoma[Title/Abstract])) OR (Sarcoma, Osteogenic[Title/Abstract])) OR (Osteogenic Sarcomas[Title/Abstract])) OR (Sarcomas, Osteogenic[Title/Abstract]))

AND

(((lung) OR (Pulmonary))))

AND

((((((Metastase) OR (Metastases)) OR (Metastasis)) OR (Metastases, Neoplasm)) OR (Metastasis, Neoplasm)) OR (Neoplasm Metastases)))

AND

((((((((((((((((((((Surgical Procedures, Operative) OR (Operative Procedures)) OR (Operative Procedure)) OR (Procedure, Operative)) OR (Procedures, Operative)) OR (Operative Surgical Procedure)) OR (Operative Surgical Procedures)) OR (Procedure, Operative Surgical)) OR (Procedures, Operative Surgical)) OR (Surgical Procedure, Operative)) OR (Surgical Procedures)) OR (Procedures, Surgical)) OR (Procedure, Surgical)) OR (Surgical Procedure)) OR (Surgery, Ghost)) OR (Ghost Surgery)) OR (Surgical)) OR (resection)) OR (Metastasectomy)) OR (Metastasectomies))

**Web of Science:**

#1: ALL=(osteosarcoma) OR ALL=(Osteosarcomas) OR ALL=(Osteogenic Sarcoma) OR ALL=(Osteosarcoma Tumor) OR ALL=(Osteosarcoma Tumors) OR ALL=(Tumor, Osteosarcoma) OR ALL=(Tumors, Osteosarcoma) OR ALL=(Sarcoma, Osteogenic) OR ALL=(Osteogenic Sarcomas) OR ALL=(Sarcomas, Osteogenic)

#2: ALL=(Metastase) OR ALL=(Metastases) OR ALL=(Metastasis) OR ALL=(Metastases, Neoplasm) OR ALL=(Metastasis, Neoplasm) OR ALL=(Neoplasm Metastases)

#3: ALL=(lung) OR ALL=(Pulmonary)

#4: ALL=(Surgical Procedures, Operative) OR ALL=(Operative Procedures) OR ALL=(Operative Procedure) OR ALL=(Procedure, Operative) OR ALL=(Procedures, Operative) OR ALL=(Operative Surgical Procedure) OR ALL=(Operative Surgical Procedures) OR ALL=(Procedure, Operative Surgical) OR ALL=(Procedures, Operative Surgical) OR ALL=(Surgical Procedure, Operative) OR ALL=(Surgical Procedures) OR ALL=(Procedures, Surgical) OR ALL=(Procedure, Surgical) OR ALL=(Surgical Procedure) OR ALL=(Surgery, Ghost) OR ALL=(Ghost Surgery) OR ALL=(Surgical) OR ALL=(resection) OR ALL=(Metastasectomy) OR ALL=(Metastasectomies)

#5: #1 AND #2 AND #3 AND #4

**Embase:**

('osteosarcoma':ti,ab,kw OR 'osteosarcomas':ti,ab,kw OR 'osteogenic sarcoma':ti,ab,kw OR 'osteosarcoma tumor':ti,ab,kw OR 'osteosarcoma tumors':ti,ab,kw OR 'tumor, osteosarcoma':ti,ab,kw OR 'tumors, osteosarcoma':ti,ab,kw OR 'sarcoma, osteogenic':ti,ab,kw OR 'osteogenic sarcomas':ti,ab,kw OR 'sarcomas, osteogenic':ti,ab,kw)

AND

('lung' OR 'pulmonary')

AND

('metastase' OR 'metastases' OR 'metastasis' OR 'metastases, neoplasm' OR 'metastasis, neoplasm' OR 'neoplasm metastases')

AND

('surgical procedures, operative' OR 'operative procedures' OR 'operative procedure' OR 'procedure, operative' OR 'procedures, operative' OR 'operative surgical procedure' OR 'operative surgical procedures' OR 'procedure, operative surgical' OR 'procedures, operative surgical' OR 'surgical procedure, operative' OR 'surgical procedures' OR 'procedures, surgical' OR 'procedure, surgical' OR 'surgical procedure' OR 'surgery, ghost' OR 'ghost surgery' OR 'surgical' OR 'resection' OR 'metastasectomy' OR 'metastasectomies')

**Cochrane library :**

#1: MeSH descriptor: [Osteosarcoma] explode all trees

#2: MeSH descriptor: [Neoplasm Metastasis] explode all trees

#3: MeSH descriptor: [Lung] explode all trees

#4: MeSH descriptor: [Surgical Procedures, Operative] explode all trees

#5: MeSH descriptor: [Metastasectomy] explode all trees

#6: (Pulmonary):ti,ab,kw (Word variations have been searched)

#7: (Surgical):ti,ab,kw OR (resection):ti,ab,kw (Word variations have been searched)

#8: #3 OR #6

#9: #4 OR #5 OR #7

#10: #1 AND #2 AND #8 AND #9

**Supplementary Appendix: Figure S1**

**Figure S1.** Sensitivity and publication bias analyses for pooled survival outcomes combining PRS and PMOS. (A–C–E) Complete versus residual resection: (A) forest plot, (C) funnel plot, (E) leave-one-out sensitivity analysis. (B–D–F) Bilateral versus unilateral metastases: (B) forest plot, (D) funnel plot, (F) leave-one-out sensitivity analysis.

**Supplementary Appendix: Figure S2**


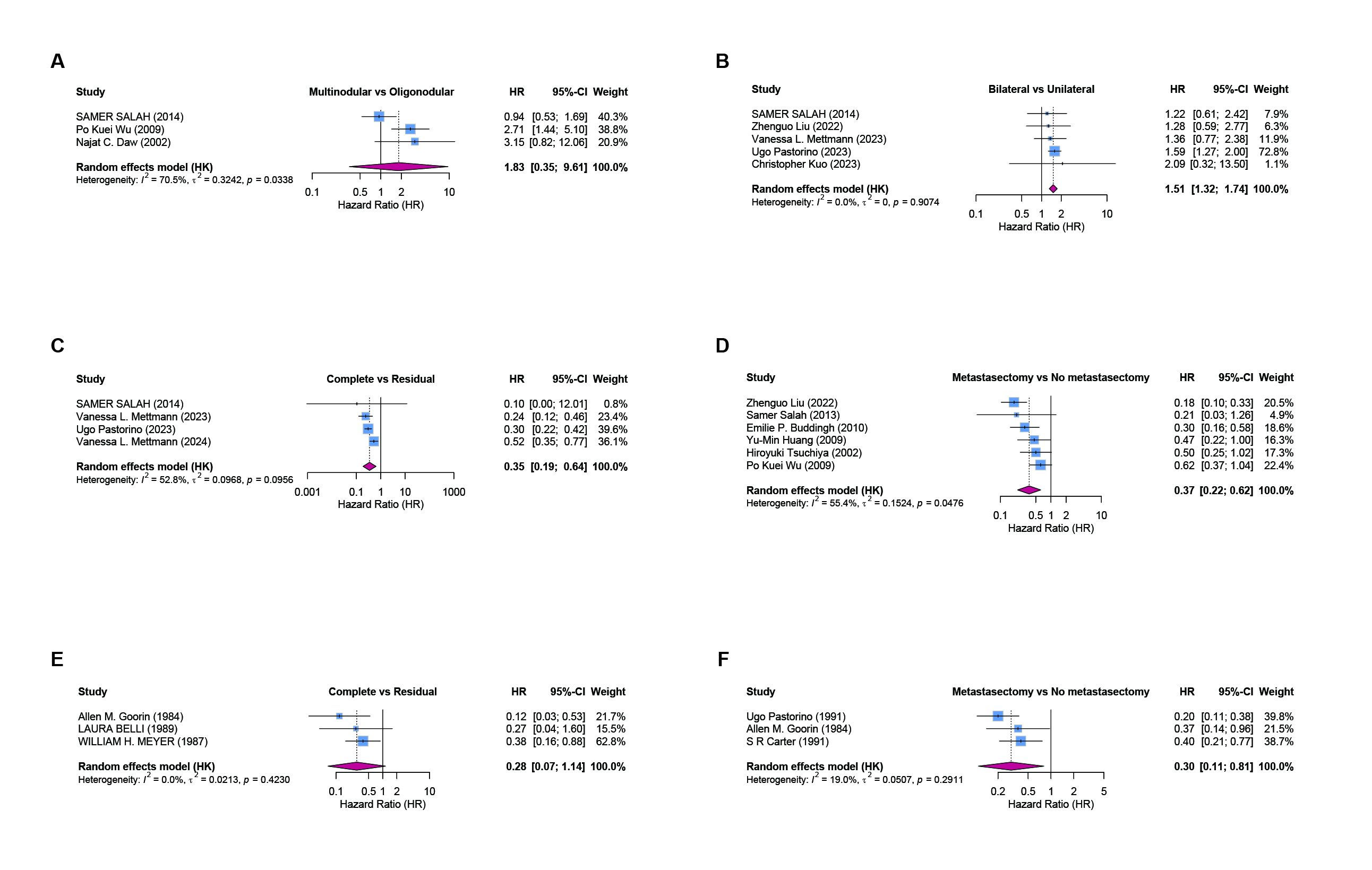


**Figure S2.** Forest plots of pooled hazard ratios for survival outcomes associated with pulmonary metastasectomy and surgery-related prognostic factors, according to publication period. (A) Oligonodular versus multinodular disease, using 3 nodules as the cutoff (studies published after 2000). (B) Bilateral versus unilateral metastases (studies published after 2000). (C) Complete versus residual resection (studies published after 2000). (D) Metastasectomy versus no metastasectomy (studies published after 2000). (E) Complete versus residual resection (studies published before 2000). (F) Metastasectomy versus no metastasectomy (studies published before 2000).

| **Supplementary Appendix: Table S1. Quality assessment** | | | | | | | | | | | | |
| --- | --- | --- | --- | --- | --- | --- | --- | --- | --- | --- | --- | --- |
|  | **Selection** | | | | | **Comparability** | **Outcome** | | | | |  |
| Assessment criteria | Representativeness of the Exposed Cohort | Selection of the Non-Exposed Cohort | Ascertainment of Exposure | Demonstration that Outcome of Interest Was Not Present at Start of Study | Comparability of Cohorts on the Basis of the Design or Analysis | | | Assessment of Outcome | | Was Follow-up Long Enough for Outcomes to Occur | Adequacy of Follow-up of Cohorts | Total |
| Samer Salah (2013) | ★ | ★ | ★ | ★ | ★ ☆ | | | ★ | ★ | | ★ | 8 |
| Hiroyuki Tsuchiya (2002) | ★ | ★ | ★ | ★ | ☆ ☆ | | | ★ | ★ | | ☆ | 6 |
| Ugo Pastorino (1991) | ★ | ★ | ★ | ★ | ☆ ☆ | | | ★ | ★ | | ★ | 7 |
| S R Carter (1991) | ★ | ★ | ★ | ★ | ☆ ☆ | | | ★ | ★ | | ☆ | 6 |
| Zhenguo Liu (2022) | ★ | ★ | ★ | ★ | ★ ★ | | | ★ | ★ | | ★ | 9 |
| Allen M. Goorin (1984) | ★ | ★ | ★ | ★ | ☆ ☆ | | | ★ | ★ | | ☆ | 6 |
| Yu-Min Huang (2009) | ★ | ★ | ★ | ★ | ★ ☆ | | | ★ | ★ | | ☆ | 7 |
| Po Kuei Wu (2009) | ★ | ★ | ★ | ★ | ★ ☆ | | | ★ | ★ | | ☆ | 7 |
| Emilie P. Buddingh (2010) | ★ | ★ | ★ | ★ | ★ ☆ | | | ★ | ★ | | ☆ | 7 |
| LAURA BELLI (1989) | ★ | ★ | ★ | ★ | ☆ ☆ | | | ★ | ★ | | ☆ | 6 |
| G. L. van Rijk-Zwikker (1991) | ★ | ★ | ★ | ★ | ★ ☆ | | | ★ | ★ | | ☆ | 7 |
| Vanessa L. Mettmann (2023) | ★ | ★ | ★ | ★ | ★ ★ | | | ★ | ★ | | ☆ | 8 |
| Ugo Pastorino (2023) | ★ | ★ | ★ | ★ | ★ ★ | | | ★ | ★ | | ★ | 9 |
| WILLIAM H. MEYER (1987) | ★ | ★ | ★ | ★ | ★ ☆ | | | ★ | ★ | | ☆ | 7 |
| Joe B. Putnam (1983) | ★ | ★ | ★ | ★ | ★ ☆ | | | ★ | ★ | | ☆ | 7 |
| SAMER SALAH (2014) | ★ | ★ | ★ | ★ | ☆ ☆ | | | ★ | ★ | | ☆ | 6 |
| Vanessa L. Mettmann (2024) | ★ | ★ | ★ | ★ | ★ ☆ | | | ★ | ★ | | ☆ | 7 |
| Timothy B. Lautz (2020) | ★ | ★ | ★ | ★ | ★ ☆ | | | ★ | ★ | | ☆ | 7 |
| Christopher Kuo (2023) | ★ | ★ | ★ | ★ | ☆ ☆ | | | ★ | ★ | | ★ | 7 |
| Allen M. Goorin (1984) | ★ | ★ | ★ | ★ | ★ ☆ | | | ★ | ★ | | ★ | 8 |
| Najat C. Daw (2005) | ★ | ★ | ★ | ★ | ☆ ☆ | | | ★ | ★ | | ☆ | 6 |
| Matthew T. Harting (2006) | ★ | ★ | ★ | ☆ | ★ ☆ | | | ★ | ★ | | ☆ | 7 |

**Supplementary Appendix: Table S2. Surgical indication**

| First author (year) | Surgical indication |
| --- | --- |
| Samer Salah (2013) [16] | NA |
| Hiroyuki Tsuchiya (2002) [17] | NA |
| Ugo Pastorino (1991) [18] | whenever technically feasible, were undertaken |
| S R Carter (1991) [19] | All patients who developed metastases confined to the lungs were considered for resection |
| Zhenguo Liu (2022) [5] | All lung lesions anatomically resectable; surgery tolerable; other metastases removable or stably controlled |
| Allen M. Goorin (1984) [20] | NA |
| Yu-Min Huang (2009) [21] | Controlled primary tumor; no extrapulmonary metastasis; adequate residual pulmonary function |
| Po Kuei Wu (2009) [22] | Thoracotomy unless lesions are unresectable or patient unfit |
| Emilie P. Buddingh (2010) [23] | Resectable metastases and no contraindications to surgery |
| Laura Belli (1989) [24] | Positive radiologic diagnosis; no primary/extra-pulmonary relapse; resectable metastases |
| G. L. van Rijk-Zwikker (1991) [25] | NA |
| Vanessa L. Mettmann (2023) [26] | Complete surgical remission after primary therapy; solitary pulmonary metastasis at first recurrence |
| Ugo Pastorino (2023) [27] | whenever technically feasible, were undertaken |
| William H. Meyer (1987) [28] | Thoracotomy for all unless nodules are too numerous/unresectable |
| Joe B. Putnam (1983) [29] | Complete resectability required; generally not resectable if >16 nodules |
| Samer Salah (2015) [30] | NA |
| Vanessa L. Mettmann (2024) [31] | NA |
| Timothy B. Lautz (2021) [32] | NA |
| Christopher Kuo (2023) [33] | NA |
| Najat C. Daw (2006) [34] | NA |
| Matthew T. Harting (2005) [35] | whenever technically feasible, were undertaken |

NA: not available.
